# Hydrothermal Sterilization Improves Initial Osteoblast Responses on Sandpaper-Polished Titanium

**DOI:** 10.3390/ma10070812

**Published:** 2017-07-17

**Authors:** Xingling Shi, Lingli Xu, Qingliang Wang, Sunarso, Lin Xu

**Affiliations:** 1 School of Materials Science and Engineering, Jiangsu University of Science and Technology, Zhenjiang 212003, China; xulingli311@hotmail.com; 2 School of Materials Science and Engineering, China University of Mining and Technology, Xuzhou 221116, China; wql889@cumt.edu.cn; 3 Department of Dental Material Science and Technology, Faculty of Dentistry, Padjadjaran University, Bandung 40132, Indonesia; sunarsochemugm@gmail.com; 4 School of Mechanical Engineering, Jiangsu University, Zhenjiang 212013, China; fgmnxl@163.com

**Keywords:** titanium implant, surface contamination, sterilization, hydrothermal, osteoblast

## Abstract

Hydrocarbon contamination accumulated on titanium (Ti) implant surfaces during storage and sterilization is unavoidable and difficult to remove. It impairs the bioactivity of implants, restricts initial interactions between implants and the surrounding biological environment, and has become a common challenge for Ti implants. To overcome this problem, sterilization was considered as the final surface modification and a novel method, hydrothermal sterilization (HS), was proposed. Briefly, stored sandpaper-polished Ti specimens were sterilized in a glass container with pure water at 121 °C for 20 min and kept in the same water until utilization. As a control, another group of specimens was sterilized with conventional autoclaving (AC) at 121 °C for 20 min and stored in sterilization pouches after being dried at 60 °C. Compared with AC, HS deposited numerous nano-sized particles on the substrates, reduced the atomic percentage of the surface carbon, and transformed the Ti surface to a super hydrophilic status. HS also increased the attachment rate, spread, proliferation, and the mineralized nodule areas of rat bone marrow-derived osteoblasts. These results suggest that HS enhances the bioactivity of Ti implants for osteoblasts, and that this biofunctionalization was attributed to nanostructure construction, hydrophilic conversion, and the effective removal of hydrocarbons. Hydrothermal sterilization is proposed to be used as a universal sterilization method for all kinds of titanium implants without apatite coating.

## 1. Introduction

Titanium has been used as raw material for dental implants for over 40 years because of its chemical inertness, outstanding biocompatibility, and mechanical strength suitable to match with natural bone [1]. Nevertheless, since the very beginning of its orthopedic application, surface modification was imposed to improve osseointegration under the expectations of the material engineer and clinician. Various techniques, including apatite coating, topology reconstruction, and chemical modulation have been studied [2,3,4]. Nowadays, it seems that the development of novel surface modification methods with sophisticated procedures has become the trend.

However, recently, some simple measurements after production have enhanced the bioactivity of classical implants with a pleasant surprise. These studies implied that the osseointegration of Ti implants without apatite coating could be further improved, with great potential. For instance, NaCl solution storage, ultraviolet (UV) irradiation, and plasma treatment of rough surfaces, such as sandblast and acid-etched (SLA) and only acid-etched treatments, have greatly improved their osseointegration [5,6,7]. These cases also drew people’s attention to the fact that we might have designed implant with a promising surface, but never prepared it for high-efficiency performance immediately before implantation. Accumulated carbon-containing contamination, wettability degradation during storage, and improper sterilization will severely impair the osteoconductivity of implants [6,8]. Unfortunately, all kinds of non-apatite-coated Ti implants are confronted with such an issue [9]. Therefore, strategies for creating a favorable initial surface status of implants immediately before implantation are worth studying.

Among all of the handling steps, sterilization is the last for both aseptically packaged and non-aseptically packaged implants. It induces physical and chemical changes on implant surfaces, depending on the specific method. The effects of these changes on cell and bone tissue responses in the initial stage were noticed by Kasemo and Stanford et al. more than 20 years ago, and were considered to have a profound influence on the bone remolding [10,11]. However, similar opinions have rarely been reported since then, because major efforts were made for ingenious surface modification methods. It is noted that, in the existing literature, sterilization methods for the same kind of implant were hardly consistent and researchers usually employed a method according to their convenience and facility. Such a situation made it difficult to compare results from different groups accurately [8,12,13]. 

As discussed above, the contradiction between storage and bioactivity degradation, as well as the uncertain and even negative influences of sterilization, have created a dilemma for current titanium implants. However, if we consider sterilization as the final surface modification and use a well-chosen method, the dilemma may be solved. Previously, we have modified the Ti surface for better osteoconductivity by hydrothermal treatment over 200 °C. However, recently we found that the surface of titanium nitride (TiN) could be decontaminated through low temperature hydrothermal treatment at 120 °C, after which the cell responses were improved [14,15]. Inspired by this finding, we proposed hydrothermal sterilization (HS) and tested this method on long-term stored SLA surfaces, acquiring promising results both in vitro and in vivo [16]. It seems that this new method can sterilize stored Ti implants reliably in addition to improving their bioactivity.

Besides those with rough surfaces, endousseus implants with relatively smooth surfaces are also widely used in some countries and regions due to their simple producing procedure, lower cost, and particular needs. Therefore, in this work we applied our novel sterilization method to sandpaper-polished Ti in order to further explore the potential applications of this method. Physiochemical properties and initial osteoblast responses on HS-treated specimens were evaluated. Traditional autoclaving (AC) was used for comparison.

## 2. Results

### 2.1. Surface Morphology and Roughness

**Figure 1:**
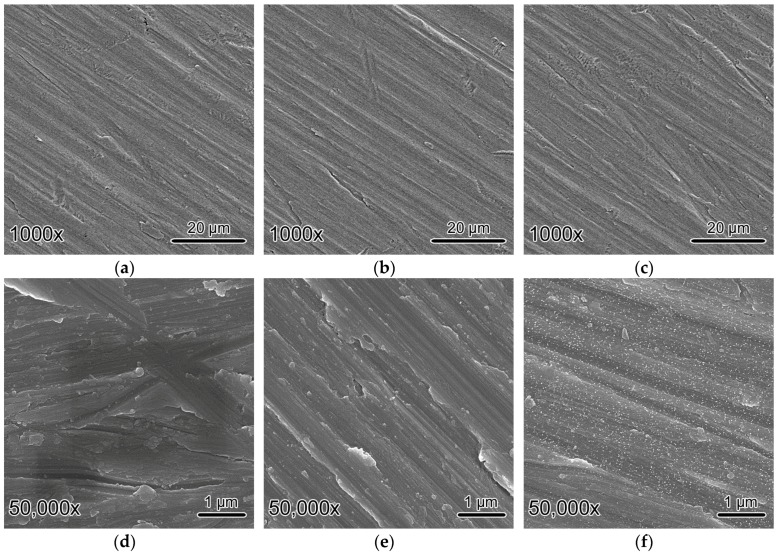
Surface morphology of (**a**) Control; (**b**) Autoclaving (AC); (**c**) Hydrothermal sterilization (HS); (**d**–**f**), magnified (a–c), respectively.

Figure 1 shows the surface morphologies of specimens under SEM observation. A lower magnification observation of 1000× revealed an identical micron-scaled topography on all types of specimens, which featured scratches from wet-abrasion during sandpaper polishing. At a higher magnification of 50,000×, changes were not found on AC specimens, whereas numerous scattering nano-sized particles (20 nm, approximately) were found on HS-treated specimens. Nevertheless, all specimens showed similar surface roughness. The *R_a_* values for the control, AC, and HS specimens were 0.32, 0.30, and 0.31 μm, respectively. Namely, the formation of nanoparticles did not affect the surface roughness significantly in the micron scale.

### 2.2. Surface Chemical Compositions

**Figure 2:**
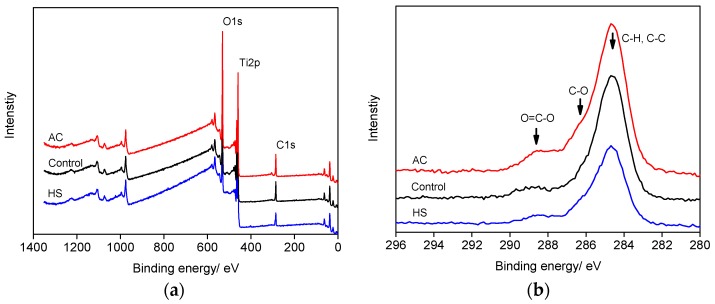
X-ray photoelectron spectroscopy survey scans (**a**) and C1s scans (**b**) of different specimens.

Table 1 shows the surface element ratio calculated from X-ray photoelectron spectroscopy (XPS) spectra. Ti, O, and C were the major elements on the surface of all three types of specimens. After four weeks of storage, the C ratio on the polished specimens was high. After AC, the C ratio increased significantly whereas Ti and O decreased. On the contrary, surface C was greatly reduced and Ti, O increased after HS. Figure 2a shows the XPS survey spectra of different samples. As shown, the intensity of C1s peak decreased apparently on the HS specimen. Figure 2b shows the high resolution C1s spectra of different surfaces. The peak could be decomposed into three components; those centered at 284.8 eV, 288.4 eV, and 288.1 eV were assigned to C−H/C−C, C−O, and O−C=O bonding, respectively. HS effectively removed carbon contamination with both low and high binding energy.

### 2.3. Wettability

**Figure 3:**
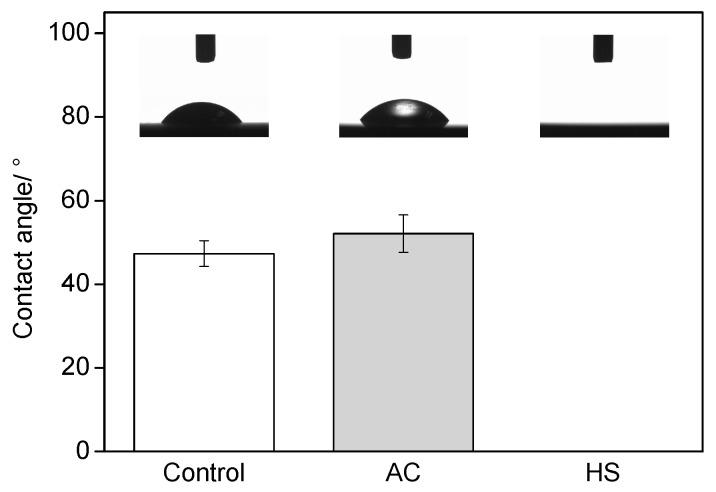
Water contact angles on different specimens.

**Figure 4:**
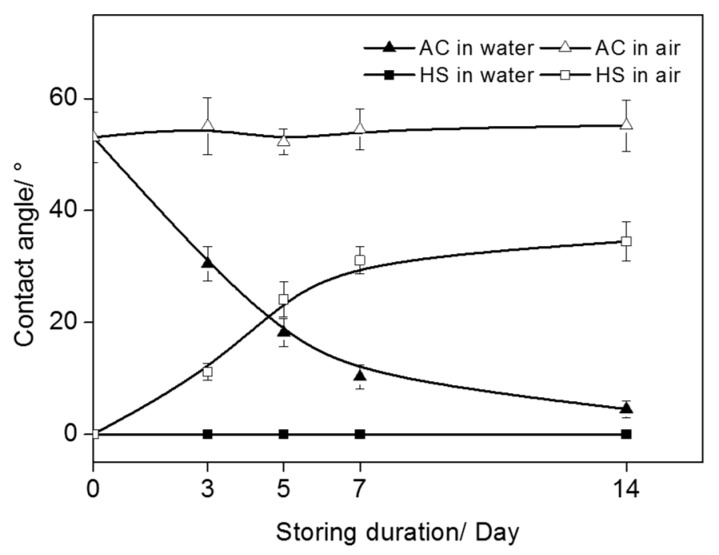
Changes of contact angle on specimens stored under different conditions.

After being stored in a poly ethylene (PE) bag for four weeks, the contact angle on the polished Ti surface was 46°. After AC treatment and drying, the water contact angle increased to 53°, as shown in Figure 3. In contrast, the water drops spread out quite fully on HS specimens and the contact angle was as low as 0°. However, the effect of storage condition on time-related changes of surface wettability was obvious. As can be seen from Figure 4, the initial superhydrophilicity of HS specimens was quickly impaired in the atmosphere, whereas the specimens kept in water remained superhydrophilic after two weeks of storage. The wettability of AC specimens could be improved when kept in water; however, the process was slow as it took about two weeks for the contact angle to reduce to less than 5°.

### 2.4. Cell Attachment

**Figure 5:**
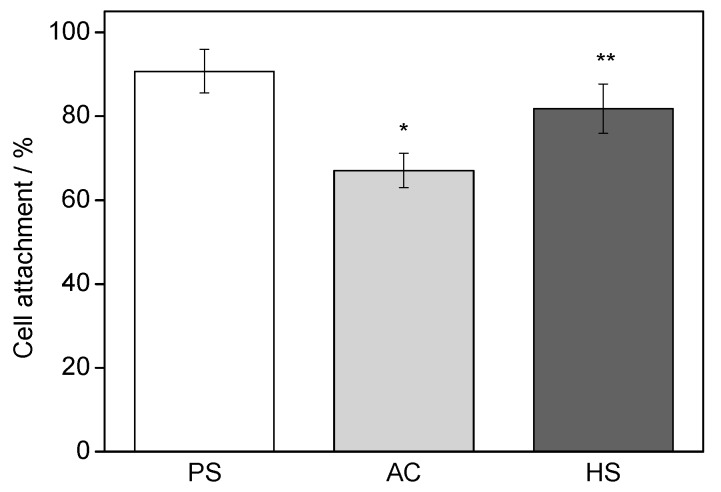
Cell attachment ratio on different specimens 3 h after seeding. * Significantly different compared with PS; ** Significantly different compared with AC.

Quantified results of cell attachment on AC and HS specimens are presented in Figure 5. After 3-h cultures, HS specimens showed significantly higher (*p* < 0.01) cell attachment compared with autoclaved ones. 

### 2.5. Cell Spreading

**Figure 6:**
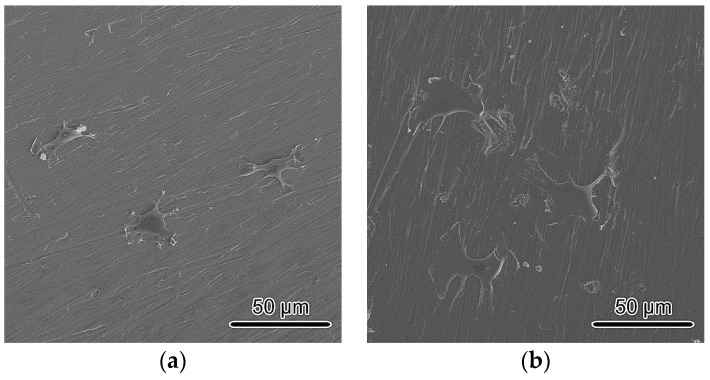
Morphologies of cells on (**a**) AC; (**b**) HS specimens 3 h after seeding.

Figure 6 presents the SEM images of cells on AC and HS specimens after 3 h of incubation. Their morphologies were quite different depending on the substrates. On AC specimens, cells presented relatively round or oval configuration and their matrix did not spread extensively. On the other hand, cells on HS specimens showed polygonal shapes and had spread out fully, occupying large areas, indicating a firm contact with the substrate. The lamellipodia was also much better developed compared with that on AC specimens, implying that the spreading was more progressive and cells were more active on HS specimens. 

### 2.6. Cell Proliferation (Viability Assessment)

**Figure 7:**
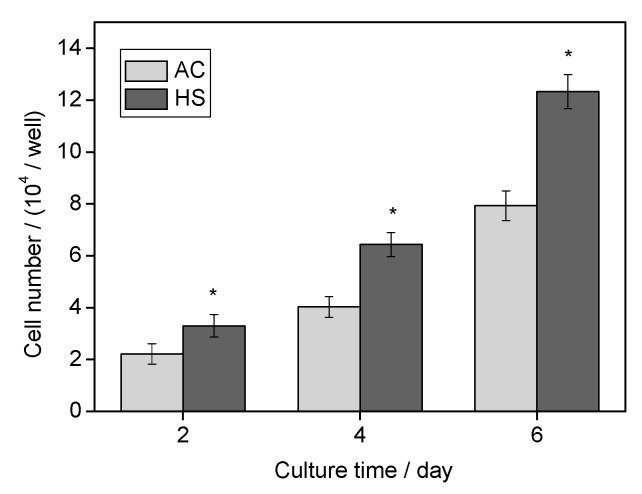
Cell proliferative activity of osteoblasts on different specimens. * Significantly different compared with AC.

Cell proliferation assayed by Alamar Blue is shown in Figure 7. During the testing period, osteoblasts proliferated continuously; however, their number varied significantly at each testing time point depending on the different sterilization methods. Cells on HS surfaces showed statistically higher proliferative activity than those on the AC surfaces at all test points (*p* < 0.05). 

### 2.7. Alkaline phosphatase (ALP) Activity

**Figure 8:**
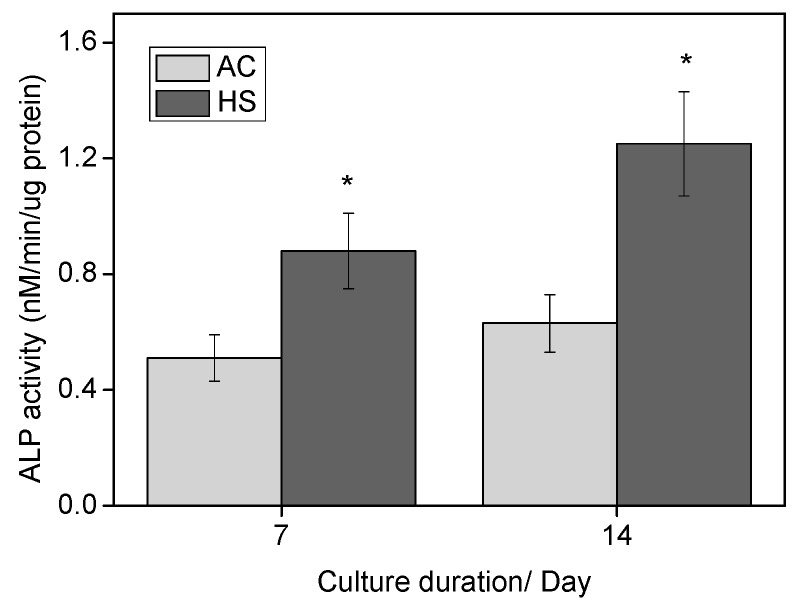
Alkaline phosphatase activities of cells on AC and HS specimens after seven and 14 days of culture. * Significantly different compared with AC.

Figure 8 shows the ALP activities of cells cultured on the surface of AC and HS specimens. After seven days of culture, ALP activity was significantly higher on HS specimens compared with that on AC specimens. After 14 days culture, ALP activity on both kinds of specimens increased, and significant difference could also be observed between the two groups.

### 2.8. Bone-Like Nodule Formation

**Figure 9:**
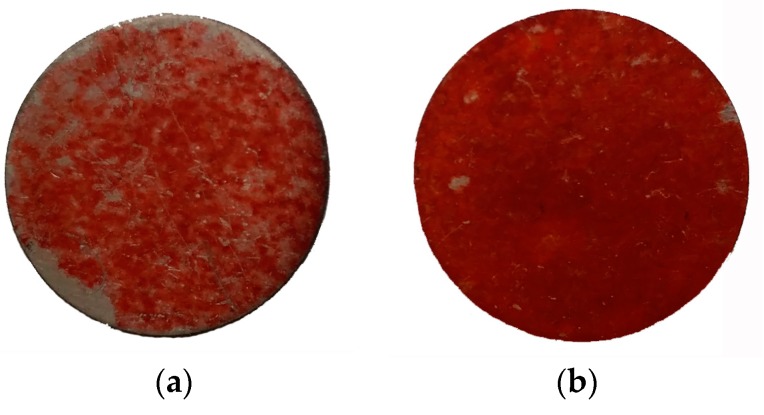
Osteoblast mineralization on (**a**) AC and (**b**) HS specimens.

Figure 9 shows the Alizarin Red S staining results of different specimens after 21 days of culture. As shown, cell mineralization had progressed on both kinds of specimens at the testing time point. However, many blank areas could be observed on AC surfaces, whereas cell calcification took place homogenously on HS surfaces, and bone-like nodules had fully covered the surface. The mineralized area on HS surfaces was significantly larger than that on AC surfaces.

## 3. Discussion

In this study, the effects of hydrothermal sterilization on in vitro osteoconductivity of sandpaper-polished Ti were proved to be substantial, and the osteoblastic phenotype was greatly enhanced as evidenced by the results of ALP activity and mineralized nodules assays. Such a phenomenon seems to have arisen from the augmented cell attachment, enhanced cell spreading, and accelerated cell proliferation. These results once again proved that HS could be used as a universal sterilization method for Ti-based bone implants.

Previously, we reported that the micron- and submicron-scaled micromorphology of SLA surface was not changed after HS under SEM observation with a magnification of 5000× [16]. However, freshly obtained FSEM results showed that the treatment at 120 °C for 20 min was enough to change the Ti surface at the nano-scale, as shown in Figure 1. These results implied that the surface of Ti could be remodeled at the nano-scale during the HS process. We hence suggested that dissolution-precipitation happened in 120 °C pure water within 20 min; nevertheless, the reaction was milder and Ti ions could not be transferred as efficiently as under higher temperatures [17].

The nano-scaled morphology change could well explain the decontamination that occurred on HS specimens, as shown in Figure 2 and Table 1. The HS process was more efficient than UV and Ozone treatment, which usually require more than 24 h to achieve a clean Ti surface [6,18]. In stark contrast, the surface C ratio on AC specimens increased. After autoclaving, surface contamination could be worsening as we had found on the SLA surface, since C-containing molecules from the atmosphere and sterilization package would be deposited onto implants during the cooling and drying stages [10,16]. 

It has been demonstrated that a clean status was necessary for the good wettability of Ti substrates, and such a precondition was especially important for a surface with micro/nano-scale hybrid structures [16,19]. It is well accepted that if such structures were seriously contaminated, they would entrap air stubbornly and increase the contact angle, whereas, if they were clean, a superhydrophilicity could be obtained. UV and plasma treatments could impose superhydrophilicity to rough surfaces based on the decomposition of hydrocarbons molecules; however, the HS process seemed to achieve it by forming a nano-scaled structure and achieving decontamination simultaneously, Figure 3. Moreover, it could preserve the clean status for a much longer time, as Ti specimens were kept from recontamination by pure water after sterilization. The results in Figure 4 highlighted the combination of HS and wet storage. Although the AC specimens could obtain a superhydrophilic surface as well by immersion in water, it would take a much longer time and would hence hinder the subsequent steps of healing, as immediate interactions between the implant and the surrounding biological environment are very important.

Both the superhydrophilicity and nanostructure were reported to benefit the initial interactions. Hydrophilic surfaces increase the adsorption of Arg-Gly-Asp (RGD)-containing proteins and promote conformational changes that benefit cell functions, whereas nanostructures usually promote the osteoblastic phenotype. In this study, improved initial responses including attachment and spreading were observed, as shown in Figure 5 and Figure 6. Recently, initial cell spreading was thought to be an indicator for further cell activities. For example, the full spreading of cells on UV or hydrothermally treated Ti was believed to release intercellular stress and triggered a series of enhanced cell performances [6,16,20]; appropriately, high cellular matrix perimeter and aspect ratio (1.5–8) implied higher ALP activity [21]. Cell spreading on HS specimens was much more extensive compared with that on AC specimens, and this might have led to the more rapid cell proliferation, as shown in Figure 7. Both cell number and cell-cell contact would be increased as proliferation was accelerated, and thus would promote cell differentiation, such as ALP activity, as shown in Figure 8. Bone-like nodule formation was the marker of late stage differentiation, and there was a positive correlation between ALP activity and bone-like nodule formation. Therefore, the above hypothesis could be verified as the mineralization on HS specimens was significantly enhanced, Figure 9.

The underlying mechanisms at present are poorly understood, yet and there are still many challenges concerning bone-implant integration. Among them, the most puzzling is that a literature review found that the average bone-to-implant contact ratio (BIC) of integrated Ti implants without apatite coating was only 45 ± 16%, far from the ideal 100% [22]. Coincidentally, this level met the minimum bone contact fraction requirement of integrated implants, according to finite element analysis [23,24]. This coincidence may be due to either negative factors in the working environment, such as stress and micromotion, or a common bottleneck of Ti implants that inhibits higher BIC. Nevertheless, as higher BIC can be commonly achieved through the application of hydroxyapatite (HA) coating, the predicament should be ascribed to Ti implants themselves.

Currently, the vast majority of commercial Ti implants are suffering from hydrocarbons contamination, which is unavoidable and difficult to remove by conventional cleaning in the clinic. It is believed to be the universal challenge for all kinds of non-apatite-coated Ti implants and will depress the initial interactions between an implant and its surrounding biological environment [6,13,25,26]. This fact was confirmed again by systemic in vitro results in this work. Actually, after 21 days of culture, round-shaped blank areas of rat bone marrow cells (RBMcs) mineralization were observed on SLA with a hydrophobic surface that had been stored for four weeks. Interestingly, these same locations had been occupied by air bubbles in the first several hours after cell seeding [16]. Hereby, we hypothesized that a similar phenomenon may occur in vivo, that is, the cells and their passages may “remember and/or mark” those initially infertile areas on the implant surface and decline immigration and mineralization there, and this may lead to a low BIC. According to the in vivo and in vitro results acquired in our studies, we believe that the proposed HS is a promising method to solve the current predicament.

In the predicable future years, Ti will still be the most important metal for osseous implants. This work revealed a great amount of hydrocarbon contamination on simply sandpaper-polished Ti surfaces as well as its effective removal by HS. Similar phenomena had been observed on SLA surfaces. In both works, HS was considered to be the final surface modification and it provided a novel way to develop more osseointegrative Ti implants in the clinic. Thus, its synthetic influences on Ti-based implants are worth further and deeper exploration. Studies have been planned for thorough investigations, such as a quantitative histological analysis. Furthermore, based on our previous studies, bioactive elements, such as Ca, Mg, Sr, Si, and Ag etc., can be easily doped onto Ti surfaces during the HS process and more promising results from these combinations are expected.

## 4. Materials and Methods

### 4.1. Specimens Preparation and Storage

Commercially pure (Grade 2) titanium plate (Baoji Titanium Industry, Baoji, China) was used as a substrate. Specimens were cut into Φ 14 mm × 1 mm pieces by linear cutting. After removing cutting oils, specimens were polished with SiC sandpaper of 280, 400, 800, and 1000 #, successively. Then they were washed with acetone, ethanol, and ultra-pure water in an ultrasonic cleaner for 10 min each. Cleaned specimens were kept individually in an airtight PE bag in a dark box for four weeks. 

### 4.2. Sterilization

Specimens were divided into two groups and sterilization was carried out in different ways. For the first group, specimens were transferred into sterilization pouches (A.R. Medicom Inc., Lachine, QC, Canada) with clean tweezers and then thermal-sealed. For the second group, specimens were put into clean reagent glass bottles (Boro 3.3, Shuniu, Chengdu, China) and, after adding ultrapure water (18 MΩ/cm) of 10 mL per disc, the bottles were sealed with propene polymer (PP) screw caps. For safety, the water in each bottle was controlled to be less than 1/3 of the volume. Both groups were transferred into the same clinical autoclave (BXM-30R, Boxun, China) and a normal autoclaving procedure of 121 °C, 20 min was conducted. After autoclaving, specimens in pouches, coded as AC, were dried at 60 °C for 30 min and then used for characterization and cell test as soon as possible. In contrast, specimens sterilized in pure water, coded as HS, were kept in bottles and dried by pure N_2_ gas right before the surface characterizations and cell tests. Stored specimens without sterilization were used as the control in surface characterizations.

### 4.3. Surface Characterizations

For both groups, specimens were characterized immediately after drying. Surface morphology was analyzed with a field emission scanning electronic microscope (FESEM, S4800, Hitachi Co., Tokyo, Japan). Roughness was measured by a 3D laser scanning microscope (VX9000, Keyence Co., Osaka, Japan). Surface chemical composition was analyzed by X-ray photoelectron spectroscopy (XPS, ESCALAB 250Xi, Thermo Fisher Scientific Inc, Waltham, MA, USA). Surface wettability was evaluated on a contact angle meter (JC2000D, Zhong Chen Co., Shanghai, China) using the sessile drop method with 2.5 µL distilled water. HS and AC specimens were also kept either in water or in air and then contact angle changes were recorded at day 3, 5, 7, and 14 to evaluate the influences of different storage conditions. 

### 4.4. Cell Culture

Rat bone marrow cells (RBMcs) collected from the femur of a 4-week-old Sprague Dawley (SD) rat were cultured in α-medium supplemented with 15% fetal bovine serum, 50 mg/L ascorbic acid, 10 mmol/L Na-β-glycerophosphate, 10^−8^ mol/L dexamethasone, 1% penicillin, 1% streptomycin, and 0.292 mg/mL l-glutamine in a humidified atmosphere containing 5% CO_2_ at 37 °C. The medium was replaced every two days and, after seven days, they were detached and seeded onto specimens.

### 4.5. Cell Attachment, Proliferation, and Mineralization

Freshly sterilized specimens were put into 24-well plates and 1 mL cell suspension with 4 × 10^4^ cells was added into each well. After 3 h of incubation, the medium was sucked and specimens were washed with phosphate buffer saline (PBS) three times. Attached cells were then trypsinized and counted using a hemocytometer. As a control, cell attachment on the bottom of polystyrene (PS) culture wells was also assessed. 

Cells were seeded at an initial density of 4 × 10^4^ cells/well and after 3 h of culture, they were washed with PBS three times and then fixed with 3% glutaraldehyde in PBS for 30 min at 4 °C. Straight after, they were dehydrated with an ascending series of ethanol solution up to 100%. Finally, after removing ethanol with hexamethyldisilazane (HMDS), morphologies of cells attached onto the surface were observed by SEM.

For proliferation evaluation (viability assessment), 1 × 10^4^ cells were seeded onto a disc in each well and Alamar Blue assay (Biosource International, Camarillo, CA, USA) was performed after two, four, and six days of culture. At each time point, the old medium was removed and the specimens were washed once with fresh culture medium. After that, freshly mixed 10% Alamar Blue-containing culture medium was added. After incubation for another 3 h, 100 µL medium from each well was transferred to a 96-well plate and the fluorescence was measured (λex = 560 nm, λem = 590 nm). The remaining Alamar Blue-containing medium in the wells was replaced with normal culture medium and the cells were further incubated.

Alkaline phosphatase (ALP) activity was assessed by measuring the transformation of *p*-nitrophenylphosphate. Initially, 1 × 10^4^ cells were seeded and after seven and 14 days culture, then cells were collected by trypsinization and centrifugation. After lysis by diluted Triton, ALP activity was determined using a colorimetric assay kit (Wako, Osaka, Japan). Total protein amount was evaluated by BCA protein assay reagent kit (Pierce Chemical Co., Rockford, IL, USA) using the enhanced protocol. ALP activity was normalized by total protein content.

Cell mineralization was marked by Alizan Red S staining. Briefly, 1 × 10^4^ cells were seeded on to the specimens and culture medium was refreshed every two days. After 21 days, specimens were washed sufficiently with PBS and then the cells were fixed with 10% formalin-PBS solution. After removing formalin with pure water, the specimens were immersed in 1.5% Alizan Red S solution for 5 min. Finally, the unreacted dye was removed with pure water and the specimens were dried in air for photographing.

### 4.6. Statistical Analysis

For physiochemical characterization, three samples were used for each test. For cell tests, a single batch of osteoblast was prepared and three samples were used for each evaluation. A *t*-test was performed using Origin 8.5 (OriginLab, Co., Northampton, MA, USA) for individual comparisons of groups and *p* < 0.05 was considered to be statistically different.

## 5. Conclusions

Compared with autoclaving, hydrothermal sterilization (HS) in pure water at 120 °C, 20 min awarded superhydrophilicity to the sandpaper-polished titanium disks and reduced the surface carbon significantly. Substantially improved attachment, spreading, proliferation, and mineralization of rat bone marrow cells-derived osteoblast were also observed. The benefit of HS to enhance the bioactivity of Ti-based implants was confirmed. It showed the potential to be utilized as a universal sterilization method for Ti implants without apatite coating.

## Figures and Tables

**Table 1:** Element percentages at the surface of different samples.

Specimens	Element Ratio (at %)		
	O	Ti	C
Control	51.9 ± 1.8	21.7 ± 1.50	26.4 ± 1.3
AC	48.6 ± 2.1	20.1 ± 1.1	31.3 ± 1.6
HS	58.1 ± 2.5	23.8 ± 1.5	18.1 ± 1.2
